# Identification by Real-time PCR of 13 mature microRNAs differentially expressed in colorectal cancer and non-tumoral tissues

**DOI:** 10.1186/1476-4598-5-29

**Published:** 2006-07-19

**Authors:** E Bandrés, E Cubedo, X Agirre, R Malumbres, R Zárate, N Ramirez, A Abajo, A Navarro, I Moreno, M Monzó, J García-Foncillas

**Affiliations:** 1 Laboratory of Pharmacogenomics, Cancer Research Program (Center for Applied Medical Research), University of Navarra, Navarra, Spain; 2 Division of Cancer and Area of Cell Therapy and Hematology Service (Center for Applied Medical Research), University of Navarra, Navarra, Spain; 3 Department of Human Anatomy, Faculty of Medicine, University of Barcelona, Barcelona, Spain; 4 Department of Medical Oncology, Hospital Municipal Badalona, Badalona, Spain

## Abstract

MicroRNAs (miRNAs) are short non-coding RNA molecules playing regulatory roles by repressing translation or cleaving RNA transcripts. Although the number of verified human miRNA is still expanding, only few have been functionally described. However, emerging evidences suggest the potential involvement of altered regulation of miRNA in pathogenesis of cancers and these genes are thought to function as both tumours suppressor and oncogenes.

In our study, we examined by Real-Time PCR the expression of 156 mature miRNA in colorectal cancer. The analysis by several bioinformatics algorithms of colorectal tumours and adjacent non-neoplastic tissues from patients and colorectal cancer cell lines allowed identifying a group of 13 miRNA whose expression is significantly altered in this tumor. The most significantly deregulated miRNA being miR-31, miR-96, miR-133b, miR-135b, miR-145, and miR-183. In addition, the expression level of miR-31 was correlated with the stage of CRC tumor.

Our results suggest that miRNA expression profile could have relevance to the biological and clinical behavior of colorectal neoplasia.

## Background

MicroRNAs (miRNAs) are 19- to 25-nt non coding RNAs that are cleaved from 70- to 100-nt hairpin-shaped precursors [1,2]. Initial estimates, relaying mostly on evolutionary conservation, suggested there were up to 255 humans miRNAs. More recent analysis have demonstrated there are numerous non conserved humans miRNAs and suggest this number may be significantly larger. Although the precise biological are not yet fully understood, miRNAs seems to be crucial factors of diverse regulation pathways, including development, cell differentiation, proliferation and apoptosis [3-6]. Moreover, miss-regulation of miRNA expression might contribute to human disease [7-10].

A more recent link between miRNA function and cancer pathogenesis is supported by studies examining the expression of miRNA in clinical samples. Calin et al reported the first evidence and showed a down-regulation of miRNA-15 and miRNA-16 in a majority of chronic lymphatic leukemia (CLL) [11]. Then, altered miRNA expression has been reported, in lung cancer [12], breast cancer [13], glioblastoma [14], hepatocellular carcinoma [15], papillary thyroid carcinoma [16] and more recently colorectal cancer [9]. These results could indicate that miRNA may be a new class of genes involved in human oncogenesis.

Up until very recently, the most common method for quantifying miRNA was Northern blotting. Over the past year, a number of different approaches to quantify miRNAs have been described, including cDNA arrays [17,18], a modified Invader assay [19], a bead-based flow cytometric assay [20] and Real-time PCR [21]. Arrays, invader assay and bead-base miRNA expression do not amplify miRNA and thus the sensitivity is often compromised. The main advantage of real-time PCR is that is more quantitative and more sensitive that other high-throughput assays. However, it could be an important disadvantage if the number of miRNA increase as expected. In this case, Real-Time PCR will be less practical than microarrays.

In our study, we analyze by Real-Time PCR the expression of 156 mature miRNA in colorectal cancer (CRC) cell lines, tumoral and normal-paired tissues from clinical samples. CRC is one of the major causes of cancer death worldwide. At a molecular level, much progress has been made in the last two decades in the identification and characterization of the genetic changes involved in the malignant colorectal transformation process [22]. A number of molecular studies have shown that colon carcinogenesis results from an accumulation of epigenetic and genetic alterations, including activating mutations of the *K-ras *proto-oncogene and inactivating mutations of *APC *and *TP53 *tumor suppressor genes or of DNA repair genes. However, this stepwise model of colorectal tumorigenesis has been mainly validated conceptually, and there is mounting evidence that alternative genetic events may occur during colorectal carcinogenesis, sometimes preferentially, sometimes randomly, and sometimes with an overlap. miRNA expression regulation could help to identify mRNA targets associated with different colorectal carcinogenesis pathways and their role as potential therapeutic targets.

In the present study, we examined by Real-time PCR the expression of 156 mature miRNA in a panel of 16 CRC cell lines and 12 matched-pair of tumoral and non-tumoral tissues from patients. We identified a subset of 13 miRNAs differentially expressed in CRC cell lines and clinical samples.

## Results and discussion

### miRNA expression in CRC cell lines

In order to investigate miRNA differential expression in human colorectal cancer, we analyzed by real-time PCR using TaqMan MicroRNA Assay kit (Applied Biosystems), the expression of 156 mature miRNAs in total RNA extracted from 15 CRC cell lines. We compared their miRNA expression profile with those of CCD-18Co (human normal colon cell line).

It is generally accepted that gene-expression levels should be normalized by a carefully selectable stable internal control gene. However, to validate the presumed stable expression of a given control gene, prior knowledge of a reliable measure to normalize this gene in order to remove any non specific variation is required. To address this problem we assessed the normalization data using three different approaches: let-7a (a miRNA that manufacturer suggests may be useful as an endogenous microRNA control according their preliminary data across several human tissues and cell lines), 18s rRNA (the most stable housekeeping gene in our CRC samples) and global median-normalization, similar to microarray analysis.

**Figure 1:**
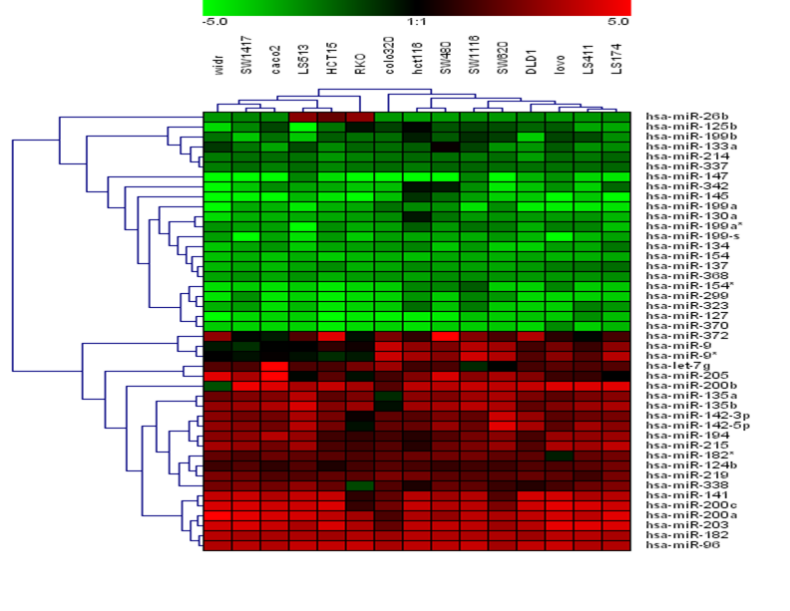
Hierarchical clustering of miRNA in CRC cell lines. 15 CRC cell lines were clustered according to the expression profile of 44 miRNAs differentially expressed and commonly detected with the three different normalization approach used between CRC and normal cell line (average linkage and Euclidean distance as similarity measure). Data from each miRNA were median centered and RQ was determined as described in material and methods. Dendrograms indicate the correlation between groups of samples and genes. Samples are in columns and miRNAs in rows. The expression values ranged from + 5 log_10 _to - 5 log_10_.

After normalization, data were transformed as log_10 _of relative quantity (RQ) of target miRNA relative to control sample. As shown Additional file 1, the different normalization approach reveals similar results. Analysis of k-means clustering (k = 3) identify a group of 22 and 22 miRNA homogeneously up-regulated and down-regulated respectively in all colorectal cancer cell lines and commonly detected with the three different normalization approach used. Figure 1 shows patterns of expression of these 44 miRNA after normalization with median-global normalization. Remarkably this classification only include those miRNA whose expression are most prominently altered and in addition the expression of this group of miRNAs is highly reproducible in all cell lines analyzed. Interestingly, clustering analysis divided CRC cell lines in two different groups. Analysis of different common genetic alterations described in colorectal cancer including activation of oncogenes (*KRAS*, *BRAF*) and inactivation of tumor supressor genes (*TP53*) and microsatellite instability status (MSI) showed that these groups could be differentiate according the presence of mutation in *KRAS *and *BRAF *genes. One group included DLD1, SW1116, SW620, SW480, HCT116, Lovo, Colo320, LS174, LS513 and LS411 CRC cell lines. All of them, except for LS411 and Colo320, harbor mutation in *KRAS *gene. On the other hand, the other group includes mainly CRC cell lines with *BRAF *mutation (WiDR, SW1417, Caco2 and RKO). SAM analysis between both groups identified 6 miRNA differentially expressed. Colorectal cancer cell lines with *KRAS *mutations showed an over-expression of miR-9, miR-9*, miR-95, miR-148a, miR-190 and miR-372, in relation to the human normal colon cell line. This over-expression was lower in whose colorectal cancer cell lines with mutations in *BRAF*. The presences of both mutations were mutually excluding. It is interesting to note that the predicted miRNA for *BRAF *regulation (using miRANDA, TargetScan and PicTar algorithms) included miR-9. This miRNA was just over-expressed in CRC cell lines with *BRAF *wild-type. Moreover, miR-372 has been recently described as potential oncogene that collaborate with oncogenic *RAS *in cellular transformation [23].

In human colorectal cancers, *KRAS *mutation has been considered an early event in the development of adenomas [24]. This genetic event is more common in large adenomas than small ones, suggesting that it may be required for the activation of adenoma progression. Recently, the activation of *BRAF *has been reported to occur by somatic mutation in many human cancers, particularly in human malignant melanoma (over 60%) [25], human colorectal cancers (5–15%) [26] and a small fraction of other cancers [27,28]. The majority of the *BRAF *mutations each represent a single nucleotide change of T-A at nucleotide 1796, resulting in the change of valine to glutamic acid at codon 599 within the activation segment of *BRAF*. Although *BRAF *mutations were found in about 5–15% of colorectal carcinomas, colorectal carcinomas with *BRAF *mutations tended to be in lower clinical tumor stages. However, it has been suggested that alteration in the *BRAF *gene may cause the activation of the *RAS/RAF/MEK/ERK *pathway [29], consequently increasing cell proliferation but suppressing the inhibition of apoptosis. Differential miRNA expression between CRC samples could help to identify different mechanisms of CRC carcinogenesis associate with alterations of the *RAS/RAF/MEK/ERK *pathway.

As shown table 1, the fold-change observed in CRC cell lines in relation to CDC18Co differed between -4.5 to -1.5 log_10 _for down-regulation and 1.4 and 3.8 log_10 _for up-regulation. Some of the genes encoding miRNA that are modulated in CRC cell lines are located in determined chromosome segments, suggesting that their tumor-specific expression could be due to DNA abnormalities. In this context, we observed a preferential down-regulation in region 14q32.31 including miRNA miR-127, miR-370, miR-299, miR-154, miR-154*, miR-323, miR-134, miR-368 and miR-337. By using a computer-assisted approach, Seitz et al. [30] have identified 46 potential miRNA located in human 14q32 domain, 40 of which are organized as a large cluster. Although some of these clustered miRNA genes appear to be encoded by a single-copy DNA sequence, most of then are arranged in tandem arrays of closely related sequences.

**Table 1:** miRNA differentially expressed in CRC cell lines.

	MEAN FOLD-CHANGE (LOG_10 _RQ)	CHROMOSOME LOCALIZATION	PUTATIVE TARGETS ASSOCIATED WITH COLORECTAL CARCINOGENESIS
hsa-miR-147	-4.56	9q32.3	
hsa-miR-127	-4.27	14q32.31	
hsa-miR-145	-4.13	5q32	TGFRII, APC
hsa-miR-370	-4.05	14q32.31	BAX, AKT1
hsa-miR-299	-3.90	14q32.31	B-CATENIN, CDKN1A
hsa-miR-199a	-3.80	1q24.3	
hsa-miR-154*	-3.71	14q32.31	MLH1
hsa-miR-199-s	-3.64	19p13.2	
hsa-miR-323	-3.56	14q32.31	MSH2
hsa-miR-154	-3.55	14q32.31	
hsa-miR-134	-3.34	14q32.31	
hsa-miR-342	-3.15	14q32.2	
hsa-miR-199a*	-3.06	1q24.3	
hsa-miR-137	-3.05	1p21.3	TGFRII
hsa-miR-368	-3.03	14q32.31	
hsa-miR-130a	- 3.02	11q12.1	TGFRII
hsa-miR-214	-2.36	1q24.3	TP53, B-CATENIN, TGFRII, BAX, CDKN2B, EGFR
hsa-miR-337	- 2.25	14q32.31	CDKN2A
hsa-miR-125b	-2.20	11q24.1	VEGF, IGFRI, VEGFR
hsa-miR-199b	-2.19	9q34.11	
hsa-miR-133a	-2.11	18q11.2	BAX, K-RAS
hsa-miR-26b	-1.82	2q35	APC
hsa-miR-133b	-1.66		K-RAS
hsa-miR-296	-1.61	20q13.32	
hsa-miR-124b	1.42		MLH1
hsa-miR-338	1.63	17q25.3	
hsa-miR-9*	1.68	5q14.3	TCF4, MSH2
hsa-let-7g	1.73	3p21.2	TGFRII
hsa-miR-372	1.76	19q13.42	TGFRII, SMAD2, MLH1, AKT1
hsa-miR-182*	1.77		
hsa-miR-219	1.93	6p21.32	TGFRII
hsa-miR-205	2.21	1q32.2	K-RAS, SMAD4, MSH2, PTEN
hsa-miR-194	2.23	1q41	
hsa-miR-142-3p	2.29		APC
hsa-miR-135a	2.36	3p21.2	MSH2
hsa-miR-215	2.42	1q41	IGFRI
hsa-miR-142-5p	2.51	17q23.2	
hsa-miR-135b	2.90	1q32.1	MSH2
hsa-miR-141	3.28	12p13.31	APC, MSH2
hsa-miR-182	3.41	7q32.2	IGFRI
hsa-miR-200b	3.44	1p36.33	MLH1
hsa-miR-200c	3.64	12p13.31	MLH1, SMAD2
hsa-miR-96	3.64	7q32.2	K-RAS
hsa-miR-200a	3.73	1p36.33	MSH2
hsa-miR-203	3.77	14q32.33	

However, 14q it is not a region usually deleted in CRC cancers although their loss have been associated with disease progression and worse prognosis [31]. On the opposite, we can hypothesize that differential expression could be regulated by modulation of its transcription. We think that this hypothesis may be supported by the observation that different "isoforms" of some down-regulated and up-regulated miRNAin CRC cell lines are located in different chromosomes, and their coordinated expression might reflect the existence of a common target. The expression of mir-200a, mir-200b and mir-200c, located in two different chromosomes (1 and 12) and with a high sequence-homology, are up-regulated in all CRC cell lines. The analysis of their putative targets showed *MLH1 *and *MSH2 *as two candidate genes whose transcription could be down-regulated by miRNA.

Our findings indicate that miRNA expression patterns are closely related to characteristics of tumor derived cell lines. These patterns may either mark specific biologic characteristics or may mediate specific biologic activities important for the pathobiology of malignant tumors.

### miRNA expression in colorectal tumours and adjacent non-tumor tissues

In order to investigate whether miRNAs are differentially expressed in CRC versus normal colon tissues, we analyzed miRNA expression in 12 matched-pairs of tumoral and non-tumoral tissues. After testing three different approaches to normalize the Ct raw data in CRC cell lines, median-normalization was selected as method for clinical samples since normal distribution was not required. Meanwhile in our study in CRC cell lines no differences were found in let-7a expression between tumoral and normal cell line, recent evidences identify let7-family as differentially expressed in CRC [9] and lung cancer [5]. Moreover, global median normalization could provide results more easily comparable with those already published with microarray technology.

To identify miRNA with significantly differential expression among CRC samples, two multivariate permutation test provided in BRB-ArrayTools were performed: Class Comparison between Groups of Arrays and SAM (Significance Analysis of Microarrays). In both cases we selected paired t-test options and a FDR (False Discovery Rate) less to 10%. Fifty-nine miRNAs were significant when Class Comparison test was applied, 68 miRNA were significant using SAM test and 53 miRNA are common in both test. As expected, fold-change observed in clinical samples is less homogeneously distributed among samples that already obtained in CRC cell lines. It is not surprising regarding that patients samples are composed of mixed populations, whereas cell lines are clearly more uniform.

**Figure 2:**
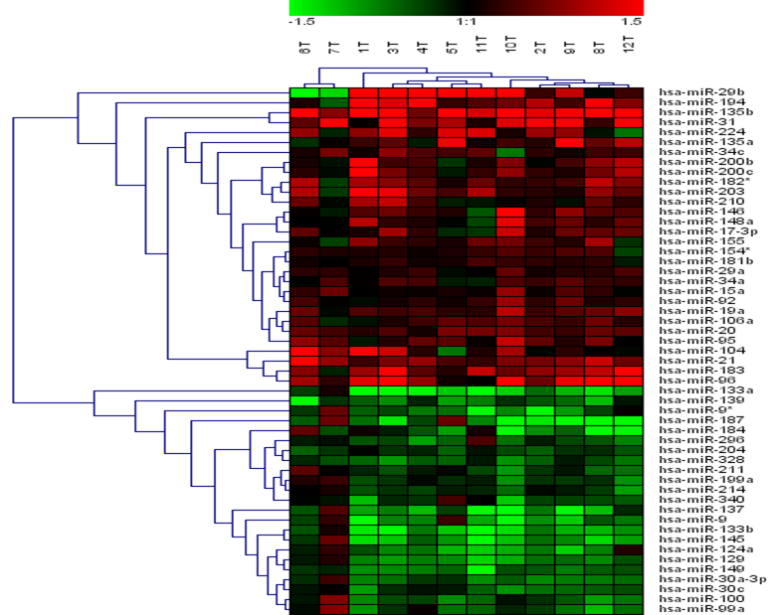
miRNA expression data from 12 CRC tumor samples. Each miRNA listed was detected as significantly differentially expressed between tumoral and paired-non-tumoral tissues with SAM and Class Comparison tests. Samples are in columns and miRNAs in rows. The expression values ranged from + 1.5 log_10 _to - 1.5 log_10_.

Interestingly, our results in CRC samples are in agreement with recent data published in CRC using direct miRNA cloning and SAGE (miRAGE). In this context, we detected an over-expression of miR-19a, miR-21, miR-29a, miR-92, miR-148a, miR-200b, and a down-regulation of miR-30c, miR-133a and miR-145 (figure 2). Moreover, change of expression of some of these miRNA has been previously reported in lung and breast cancer, B-cell lymphomas, and glioblastoma. miR-19a and miR-20 are including in the cluster miR-17-92 and it is located at intron 3 of *C13orf25*. The transfection of *C13orf25 *in lung cancer cell line enhancing cell growth and the introduction of miR-17-92 into hematopoietic stem cells in *Eu-myc *transgenic mice accelerates the formation of lymphoid malignancies. Furthermore, miR-21 has been described as an antiapoptotic factor in human glioblastoma cell lines. In contrast, other authors report that miR-21 suppression increase growth in HeLa cells without affecting their apoptosis. The different biologic effects of any particular miRNA in different cells could be dependent of the cell-specific repertoire in target genes. Some of miRNA differentially expressed in CRC samples have been associated with clinical parameters in other cancers. In particular, miR-145 is progressively down-regulated from normal breast to cancer with high proliferation index and miR-21 is progressively up-regulated with high grade tumor stage.

To identify the smallest set of predictive miRNAs differentiating normal versus cancer tissues, we have used support vector machines (SVMs) techniques. We attempted to use the class prediction tool (BRB-Array tools) that creates a multivariate predictor for determining to which of the two classes a given sample belongs. Several multivariate classification methods are available, including the Compound Covariate Predictor, Diagonal Linear Discriminate Analysis, Nearest Neighbor Predictor, Nearest Centroid Predictor, and Support Vector Machine Predictor. The classifier is composed for 18 miRNA, 10 down-regulated and 8 up-regulated, all of them significantly different by Class Comparison and SAM tests.

**Figure 3:**
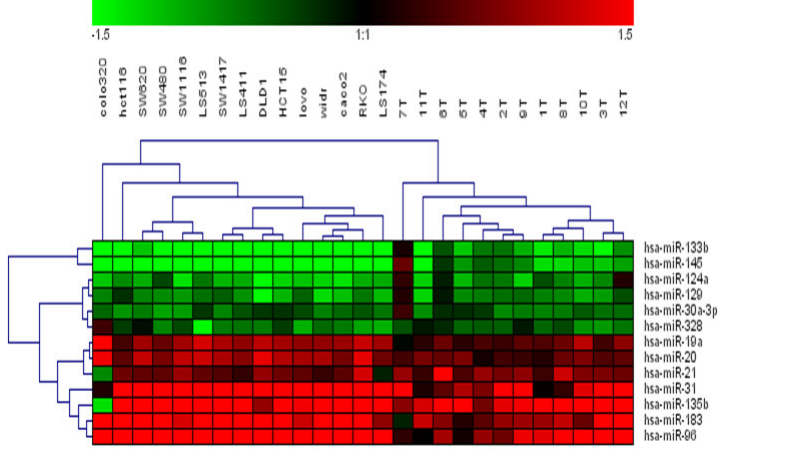
Hierachical clustering of CRC cell lines and tumor samples by using expression of 13 miRNAs that have found differentially expressed between neoplastic conditions (CRC cell lines and tumor samples) and non-tumoral colon tissues. Samples are in columns and miRNAs in rows.

When we compared expression of these miRNA in CRC cell lines, 5 of 18 miRNA were revealed in the k-means analysis as those of highest fold-changes (in relation to CDC18Co). However, Class Comparison analysis between 15 CRC cell lines and the 12 non-tumoral colon tissues identifies 13 miRNA altered in both systems, CRC patients samples and CRC cell lines (Table 2). These results could indicate that miRNA profile in CRC cell lines can not be used to infer miRNA expression in clinical samples meanwhile cell lines can be used as model to validate and performed functional assays of data obtained in clinical samples. In this sense, hierarchical clustering of expression of these 13 miRNAs in CRC patients samples and CRC cell lines clearly separate samples in two groups: in one branch, the most different of non-tumoral samples were included all the CRC cell lines, and in the other branch tumoral samples of patients (Figure 3).

The expression of 5 of 13 miRNA is already described altered in CRC, lung and breast cancer, glioblastoma, B-cell lymphoma and CLL. Among the differentially expressed miRNAs, miR-31, miR-96, miR-133b, miR-135b, miR-145 and miR-183 as the most consistently deregulated in CRC. Two of them, miR-133b and miR-145 were down-regulated and the remaining four, miR-31, miR-96, miR-135b and miR-183, were up-regulated, suggesting that they may potentially act as tumor suppressor genes or oncogenes, respectively.

miR-145 was identified as a specific miRNA down-regulated in colorectal neoplasia and analysis of their pre-miRNA indicate that this reduction is due to posttranscriptional process [32]. Recently, Cummins et al obtained similar results in CRC [9] and down-regulation of miR-145 have also reported in lung [8] and breast cancer [13]. In our study, expression of miR-145 was not detected in any of 15 CRC cell lines tested and down-regulation was detected in all tumor samples. Other important down-regulated miRNA in our study was miR-133b. In our knowledge, this miRNA has not previously identified deregulated in cancer. For both down-regulated miRNAs (miR-145 and miR-133b), it may be expected that potential targets could include oncogenes or genes encoding proteins with potential oncogenic functions. Indeed, among putative targets for miR-145 with potential oncogenic functions, Iorio et al [13] described *MYCN, FOS, YES*, and *FLI*, cell cycle promoters such as cyclins D2 and L1; and MAPK transduction proteins such as *MAP3K3 *and *MAPK4K4*. Among putative targets of *miR-133b*, the most notable oncogenic target is *KRAS*. Interestingly, the proto-oncogen *YES1 *and the transduction protein *MAP3K3 *were potential targets of both miR-145 and miR-133b.

For the up-regulated miRNAs, miR-135b, miR-31, miR-96 and miR-183, it may be expected that gene targets belong to the class of tumor suppressor genes. miR-96, miR-182 and miR-183 are located in the same chromosomal region, 7q32.2. miR-182 was not detected as preferentially over-expressed with the most restricted analysis, but their up-regulation was clearly observed in CRC cell lines analysis (table 1). *CHES1 *protein was identified as a potential target of both miR-96 and miR-182. *CHES 1 *is a member of the forkhead family of transcription factors that repress genes involved in apoptosis. Other members of this family, including *FOXF2*, *FOXK2*, *FOXO1A*, *FOXO3A *and *FOXQ*1, were also found as putative targets of miR-182, miR-183 and miR-96.

**Figure 4:**
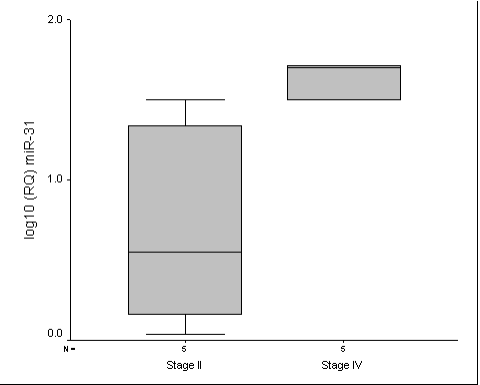
Real-time PCR analysis of miR-31 expression between stage II and stage IV tumor samples. Differences was significant after Mann Whitney U test (p = 0.028).

Finally, our analysis of a small number of CRC samples compared miRNA expression in tumors according to pathological stage (stage II versus stage IV). The up-regulation of miR-31 was significantly higher in stage IV than in CRC samples stage II (p = 0.028) (Figure 4). The expression levels of miR-31 were higher in the tumor samples and CRC cell lines in comparison to the non-tumoral samples and was related to pathological stage, suggesting that this miRNA could contribute to both, the tumorogenesis and the acquisition of a more aggressive phenotype in CRC. Other members of the forkhead family transcription factors, such as *FOXC2 *and *FOXP3*, were identified as putative targets of miR-31. Future studies will determine the correlation between of these miRNAs and their host genes in CRC.

In summary, our results by Real-time PCR identify alterations of miRNA expression in CRC that may deregulate cancer-related genes and would provide potential mechanisms that underly in the carcinogenesis and further acquisition of a more aggressive phenotype in colon cancer.

## Materials and methods

### Cell lines and tissues

The following 16 cancer cell lines were used: CDC18Co (human normal colon), HCT15, RKO, DLD1, Lovo, LS411, SW1417, Caco2, LS513, SW1116, HCT116, SW480, Colo320, SW620, WiDR and LS174. Colorectal cell lines were cultures in a humidified atmosphere of 95% air, 5% CO2 using recommended medium and 10% FBS. Colorectal tumours and paired-adjacent normal colorectal tissues were received from our Institutional Bank of Tumors. Collection and distribution of colorectal tissues were approved by the appropriate Institution Review Board.

### RNA extraction, reverse transcription and Real-Time PCR quantification

Total RNA was extracted from cells with a cell density of 75% confluent using Trizol^® ^total RNA isolation reagent (Gibco BRL, Life Technologies, Gaitherburg, MD, USA) as per the manufacturer's protocol. Total RNA was isolated from the frozen tissues disrupted using an Ultra Turrax T25 homogenizer and using Trizol. The concentration was quantified using NanoDrop Specthophotometer (NanoDrop Technologies, USA).

cDNA was synthesized from total RNA using gene-specific primers according to the TaqMan MicroRNA Assay protocol (PE Applied Biosystems, Foster City, CA). Reverse transcriptase reactions contained 10 ng of RNA samples, 50 nM stem-loop RT primer, 1 × RT buffer, 0.25 mM each of dNTPs, 3.33 U/μl MultiScribe reverse transcriptase and 0.25 U/μl RNase Inhibitor (all purchased from cDNA Archive kit of Applied Biosystems). The 7.5 μl reactions were incubated in an Applied Biosystems 9800 ThermaCycler in a 96-well plate for 30 min at 16°C, 30 min at 42°C, 5 min at 85°C and then held at 4°C.

Real-time PCR was performed using an Applied Biosystems 7300 Sequence Detection system. The 10 μl PCR included 0.67 μl RT product, 1× TaqMan Universal PCR master mix and 1 μl of primers and probe mix of the TaqMan MicroRNA Assay protocol (PE Applied Biosystems). The reactions were incubated in a 96-well optical plate at 95°C for 10 min, followed by 40 cycles of 95°C for 15s and 60° for 10 min. The Ct data was determinate using default threshold settings. The threshold cycle (Ct) is defined as the fractional cycle number at which the fluorescence passes the fixed threshold.

### Normalization and data analysis

Careful normalization is essential for the accurate quantification of mRNA levels and commonly, normalization of the target gene with an endogenous standard, mainly housekeeping genes, is applied. However for miRNA, there is not data about the expression of miRNA as normalization control.

In our study, we have tried different approach for normalization data. First, miRNA expression data was normalized to let7-a miRNA (according to the manufacturer's suggestions). Relative quantification of miRNA expression was calculated with the 2^-ΔΔCt ^method (Applied Biosystems User Bulletin N°2 (P/N 4303859)). The data were presented as log_10 _of relative quantity (RQ) of target miRNA, normalized respect to miR-let-7a and relative to a calibrator sample. As calibrator, we are used for colorectal cancer cell lines CDC18Co (human normal colon) and for CRC samples the paired-normal tissues.

The TaqMan Human Endogenous Control Plate (Applied Biosystems) precoated with lyophilized primers and probes for 11 differently commonly human control genes was also used to asses gene expression in two tumours and two normal colon tissues. PCR was set up according to the manufacturer's instructions. Among 11 genes analyzed, the variability in expression of 18s rRNA was shown to be the lowest (data not shown). Therefore, our second normalization approach showed the data as log_10 _of relative quantity (RQ) of target miRNA, normalized to 18s rRNA and relative to control sample.

Finally, similar to microarray data, raw data Ct was normalized and analyzed using BRB ArrayTools version 3.3.2. (Richard Simon and Amy Peng Lam, National Cancer Institute, Bethesda). After global median normalization, normalized data were presented as log_10 _of relative quantity (RQ) of target miRNA relative to a control sample. Class Comparison and Significant analysis of microarrays (SAM) was performed to identify differentially expressed miRNAs between tumors and normal samples.

Visualization of results was performed with the different normalized data using average linkage and Euclidean distance as a measurement of similarity using GENESIS Software (Alexander Sturn, Institute for Genomics and Bioinformatics, Graz University of Technology).

## Competing interests

The author(s) declare that they have no competing interests.

## Authors' contributions

All authors participated in the design of experiments. EB was responsible for qRT-PCR studies and drafted the manuscript. EC and XA assisted with analysis, and contributed to drafting the manuscript. RM and RZ contributed to methods development and qRT-PCR analysis. NR, AA, and AN assisted with methods development and data analysis. IM and MM provided the CRC tumor samples and assisted with critical examination of the manuscript. JGF designed and coordinated of the study, and drafted the manuscript All authors read and approved the final manuscript.

**Table 2:** miRNA differentially expressed in CRC patients samples and CRC cell lines.

	**Mean fold-change (log**_**10**_**RQ) CRC patients samples**	**Mean fold-change (log_10_RQ) CRC cell lines**	**Chromosome localization**	**Correlation with cancer**
hsa-miR-133b	-1.01	-3.38	6p12.2	
hsa-miR-145	-0.84	-4.95	5q32	↓ CRC, lung, breast cancer.
hsa-miR-129	-0.67	-0.88	7q32.1	
hsa-miR-124a	-0.64	-1.11	8p23.1	↓ lung cancer
hsa-miR-30-3p	-0.53	-0.63	6q13	
hsa-miR-328	-0.52	-0.65	16q22.1	
hsa-miR-19a	0.49	0.96	13q31.3	↑ CRC, lung cancer, B-cell lymphomas, CLL
hsa-miR-20	0.49	1.01	13q31.3	↑ lung cancer, poorly differentiated HCC
hsa-miR-21	0.78	0.48	17q23.2	↑ CRC, glioblastoma, lung, breast cancer, papillary thyroid carcinoma
hsa-miR-183	0.88	1.74	7q32.2	
hsa-miR-96	1.04	1.99	7q32.2	
hsa-miR-31	1.09	2.59	9p21.3	
hsa-miR-135b	1.60	1.78	1q32.1	

## Supplementary Material

Additional File 1 Supplementary figure 1. Analysis of k-means clustering (k = 3) of CRC cell lines identify a group of 22 and 22 miRNA homogeneously up-regulated and down-regulated respectively in CRC cell line and commonly detected with the three different normalization approach used: (a) let-7a, b)18s rRNA and c) global median-normalization. After normalization, data were transformed as log_10 _of relative quantity (RQ) of target miRNA relative to control sample (normal colon cell line). Click here for file
