# Supplementary figures and images for: Identification by Real-time PCR of 13 mature microRNAs differentially expressed in colorectal cancer and non-tumoral tissues
Source: Mol Cancer. 2006 Jul 19;5:29. doi: 10.1186/1476-4598-5-29 (PMC1550420; doi:10.1186/1476-4598-5-29)

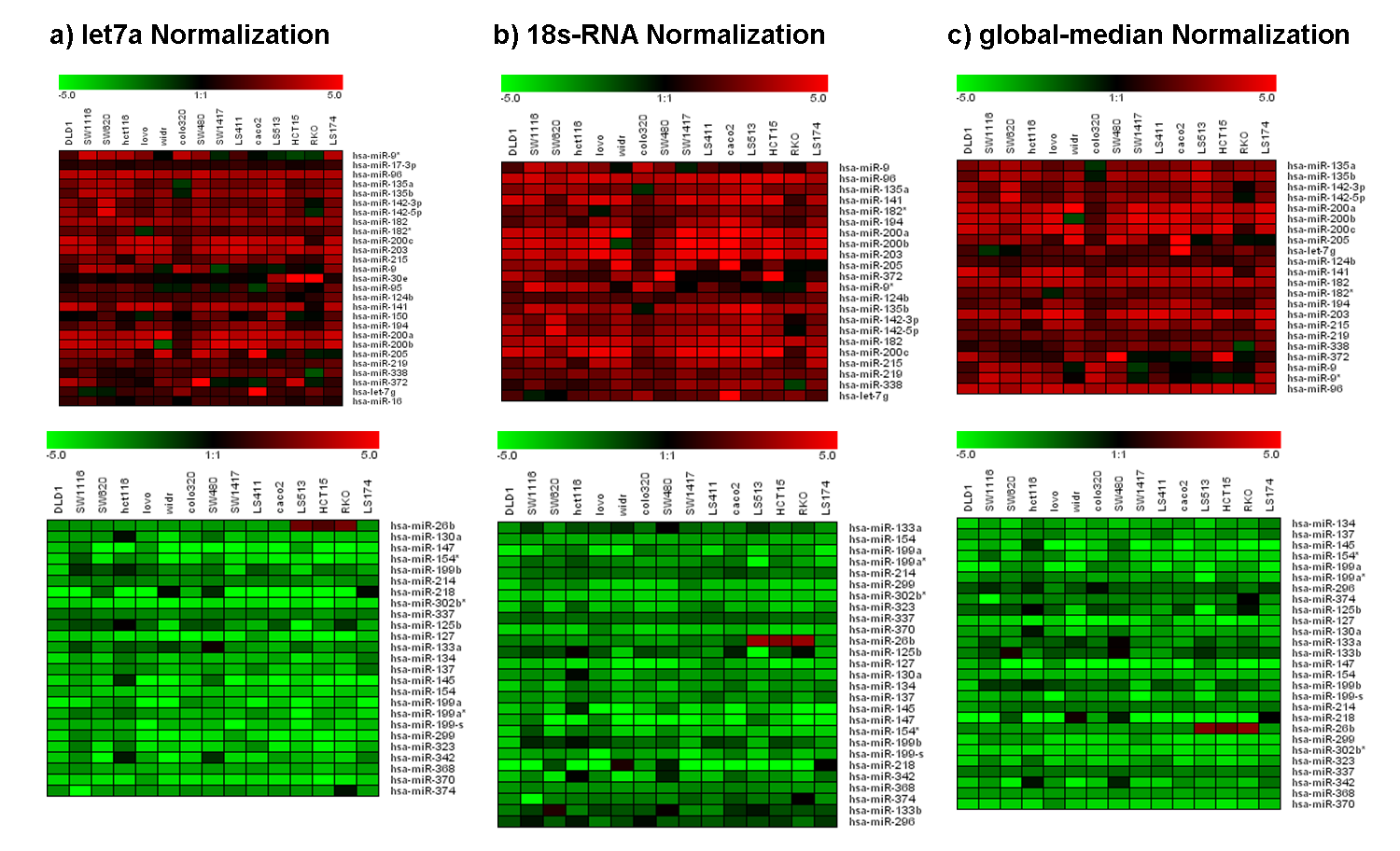

Supplement: Additional File 1 — Supplementary figure 1. Analysis of k-means clustering (k = 3) of CRC cell lines identify a group of 22 and 22 miRNA homogeneously up-regulated and down-regulated respectively in CRC cell line and commonly detected with the three different normalization approach used: (a) let-7a, b)18s rRNA and c) global median-normalization. After normalization, data were transformed as log10 of relative quantity (RQ) of target miRNA relative to control sample (normal colon cell line). [file 1476-4598-5-29-S1.tiff]
